# Characteristics and Clinical Course of Alveolar Echinococcosis in Patients with Immunosuppression-Associated Conditions: A Retrospective Cohort Study

**DOI:** 10.3390/pathogens11040441

**Published:** 2022-04-06

**Authors:** Ansgar Deibel, Cordula Meyer zu Schwabedissen, Lars Husmann, Felix Grimm, Peter Deplazes, Cäcilia S. Reiner, Beat Müllhaupt

**Affiliations:** 1 Department of Gastroenterology and Hepatology, University Hospital Zurich, 8091 Zurich, Switzerland; cordula.meyerzuschwabedissen@usz.ch (C.M.z.S.); beat.muellhaupt@usz.ch (B.M.); 2 Department of Nuclear Medicine, University Hospital Zurich, 8091 Zurich, Switzerland; lars.husmann@usz.ch; 3 Institute of Parasitology, Vetsuisse and Medical Faculty, University of Zurich, 8057 Zurich, Switzerland; felix.grimm@uzh.ch (F.G.); deplazesp@access.uzh.ch (P.D.); 4 Institute of Diagnostic and Interventional Radiology, University Hospital Zurich, 8091 Zurich, Switzerland; caecilia.reiner@usz.ch

**Keywords:** alveolar echinococcosis, *Echinococcus multilocularis*, immunosuppression-associated conditions, immunosuppression

## Abstract

Objectives: Since the change in the millennium, an increase in cases of alveolar echinococcosis (AE) has been observed in endemic European countries. Previous studies indicate that a significant proportion of the new AE cases have an immunosuppression-associated condition (IAC). The aim of the current study was to determine how IACs impact the number of new AE diagnoses per year and the characteristics of AE at diagnosis and its clinical course at our center. Methods: Retrospective analysis of 189 patients with AE diagnosed between 2000 and 2021 and participating in the Zurich Echinococcosis Cohort Study (ZECS) included clinical characteristics of AE at diagnosis and report of an IAC, as well as the clinical course during follow-up. Results: Of 189 patients participating in this study, 38 had an IAC reported at, or shortly after, AE diagnosis. Over time, there was a steeper increase in the number of newly diagnosed AE patients without an IAC than the number of patients with IAC. Patients with an IAC were older at diagnosis, more frequently had an incidental finding of AE, smaller mean lesion size, and negative Em18 serology. All but two showed favorable outcomes on the last follow-up. Conclusion: IACs have little impact on the increase in new AE cases, as well as on the extent of the disease at diagnosis and clinical course.

## 1. Introduction

Alveolar echinococcosis (AE) is an orphan zoonosis caused by the larval stage of the fox tapeworm *Echinococcus multilocularis.* The wildlife cycle involves canines as definite and predominantly rodents as intermediate hosts. Humans can acquire AE through accidental swallowing of *E. multilocularis* eggs, which hatch in the intestine and migrate primarily to the liver [1]. Less frequently, other organs, such as the lungs, kidney, spleen, and brain are also involved [1]. Alveolar echinococcosis behaves like a malignant tumor with mostly infiltrative growth and occasional metastatic spread. Without adequate treatment, 90% of patients with AE die within 10 years [2]. The mainstay of treatment is surgical resection and medical treatment with benzimidazoles [3]. However, due to the advanced disease stage at diagnosis, radical surgery is only possible in 20–50% of patients [4]. Inoperable AE requires life-long medical treatment with benzimidazoles [4], which are very efficacious in slowing parasite growth and stabilizing disease [5]. Since their introduction, the life expectancy of AE patients has increased significantly, almost reaching that of the normal population [6].

Since the change in the millennium, a two- to threefold increase in AE incidences has been observed in Switzerland, France, and Germany [7,8,9,10]. Different explanations have been discussed [11,12]. In Switzerland, the fox population has increased significantly following a successful rabies eradication in the 1980s and today colonizes densely populated areas [7]. Approximately 30–50% of the Swiss fox population is infected with adult *E. multilocularis* worms, even in urban areas, which today have extraordinarily high fox densities [13]. Through this increase in environmental contamination with *E. multilocularis* eggs especially in densely populated areas, a much higher increase in new cases could be expected after the long incubation time. A similar epidemiological situation was calculated for highly endemic areas of Germany with an approximately tenfold increase in the environmental contamination with *E. multilocularis* eggs [14]. Based on epidemiological observations, it was postulated that a probably major part of exposed individuals does not develop AE based on innate immune mechanisms interfering with oncosphere invasion and early development [11]. On the other hand, the role of acquired immunity in controlling parasite growth, if established in the liver, was well documented in experimental murine studies with evidence of a periparasitically induced immune interaction [15]. Indeed, only single “abortive” or “died-out” AE cases have been observed probably based on protective immune mechanisms.

Interestingly, in France, an observation was made indicating that, since 2002, a significant proportion of patients with a new AE diagnosis also had an immunosuppression-associated condition (IAC), possibly explaining the increase in cases since the change in the millennium [8]. Similar findings have also been reported by the study group from Bern [10]. Furthermore, severe forms of AE have been observed in patients with AIDS, pregnancy, and transplant recipients [16,17,18]. In animal models, pharmacological immunosuppression has been shown to facilitate *E. multilocularis* oncosphere invasion [19,20].

Immunosuppression, however, is a broad concept and can be caused by a vast variety of diseases, as well as a plethora of pharmaceuticals, through many different mechanisms and to various degrees. An increased risk of infection has been reported in patients with cancer [21], chronic inflammatory diseases [22,23], advanced chronic kidney disease [24], cirrhosis [25], diabetes mellitus [26], HIV/AIDS [27], and various pharmaceuticals, including immunosuppressives for organ transplantation [28]. In the current study, we determined how many patients were newly diagnosed with AE at our center during the time period 2000–2021, how many also had an immunosuppression-associated condition at or before diagnosis, and whether this was associated with differences in the characteristics of AE at diagnosis and the clinical course.

## 2. Methods

### 2.1. Study Population and Design

**Figure 1:**
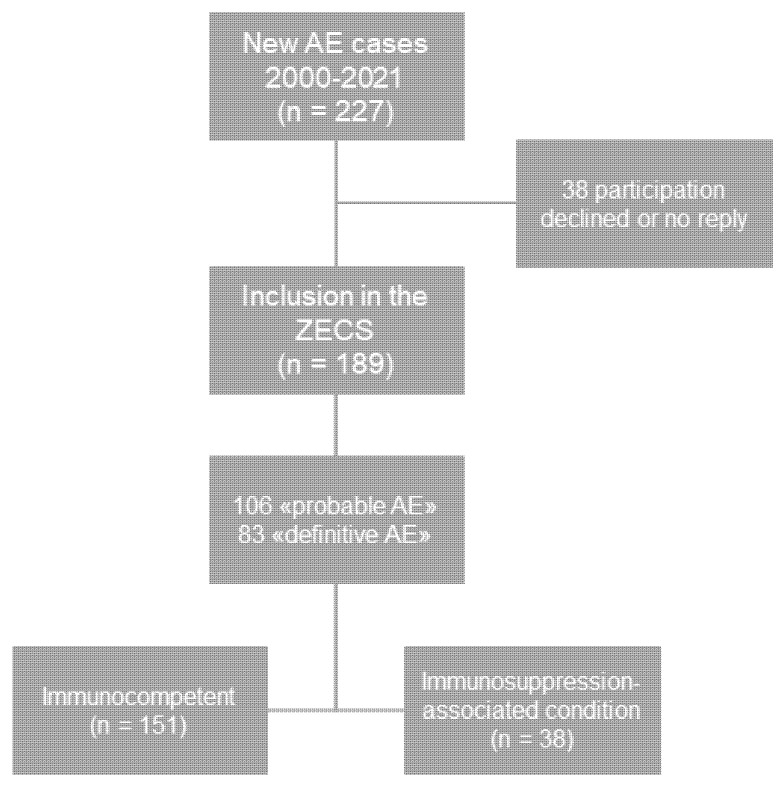
After a query of the electronic hospital information system KISIM (Cistec), 227 patients with a new diagnosis of AE during the time period 2000–2021 could be identified. All met the inclusion criteria for participation in the Zurich Echinococcosis Cohort Study. In total, 189 patients consented to participation, of which 106 met the WHO case definition of “probable AE”, and 83 met the WHO case definition of “definitive AE”. In 38 of the 189 participants, an immunosuppression-associated condition was reported at or up to 1 year after AE diagnosis. The rest was considered immunocompetent.

A query of the electronic hospital information system (KISIM, Cistec, Zurich, Switzerland) was performed to identify all patients who had been treated for alveolar echinococcosis at our center between 2000 and 2021. During that time period, 227 patients presented with a newly diagnosed AE. These patients were asked to join the cohort study during outpatient visits or, when no longer under surveillance, by letter. Patients who died before the launch of the cohort study could be included without informed consent, as permitted by the cantonal ethics committee. Patients with a typical imaging finding and positive serology for AE were considered to have a “probable diagnosis” according to the World Health Organization (WHO) case definition [29]. Those with compatible histopathology or detection of *E. multilocularis* DNA through PCR in a resectate or biopsy were considered to have a “definitive diagnosis” according to the WHO case definition [29]. Out of 227 patients, 189 consented to participation in the ZECS and represented the study population of the current study (Figure 1). 

The retrospective analysis of clinical characteristics at diagnosis included date of and age at diagnosis, gender, reported PNM classification and stage of the disease [29], anti-Em18 antibody ELISA results, clinical presentation (incidental or symptomatic), treatment (surgical resection or benzimidazole treatment) and, in case contrast-enhanced cross-sectional imaging at diagnosis was available, number of AE liver lesion and size. Additionally, patients were screened for existing immunosuppression-associated conditions (IACs) within 10 years prior to and up to 1 year after the diagnosis of AE. IACs were defined as any solid organ or hematologic/lymphoid malignancy, chronic inflammatory disease, chronic kidney disease KDIGO stage 4 and 5, cirrhosis Child–Pugh A–C, diabetes type 1 and 2, solid organ or hematopoietic stem cell transplantation, acquired or hereditary immunodeficiency syndromes, and use of immunosuppressive medication. Malignant diseases of the skin were only considered if locally advanced or metastasized to lymph nodes or other organs. Chronic inflammatory diseases included Crohn’s disease and ulcerative colitis; large vessel, small vessel, ANCA-positive, Wegener’s, Churg-Strauss, microscopic vasculitis, and Behcet’s disease; collagenosis (systemic lupus erythematodus, Sjögren’s syndrome, systemic sclerosis and mixed connective tissue disease); rheumatoid arthritis, spondylarthritis, inflammatory myositis, polymyalgia rheumatica, cryoglobulinemia, Still’s disease, and anti-phospholipid syndrome. Hereditary immunodeficiency syndromes included common-variable immunodeficiency, IgG/IgA deficiency, B-/T-cell deficiency, complement deficiency, and idiopathic immunodeficiency. Medication that was considered immunosuppressive were conventional chemotherapeutics, calcineurin inhibitors, cyclophosphamide, mycophenolate, prednisone (>3 months, ≥5 mg), azathioprine, 6-mercaptopurine, methotrexate, leflunomide, anti-TNF-α, anti-integrin, anti-CD20, anti-BAFF, anti-IL-1, anti-IL-6, anti-IL-12, anti-IL-23, and anti-CTLA4 antibodies, as well as PDE4 and JAK inhibitors.

Furthermore, in patients with AE and an immunosuppression-associated condition, the date of the last follow-up, follow-up duration, and the clinical course of AE were determined. Complete follow-up included existing current patient history, imaging report (US, CT, or MRI), serology, and blood analysis until last contact or study closure date by 31 December 2021. Progression of AE was defined as new lesions and/or an increase in the size of known lesions on imaging.

This study is reported following the Strengthening the Reporting of Observational Studies in Epidemiology (STROBE) Statement Checklist.

### 2.2. AE Serologic Testing

All patients had serologic testing for AE performed to verify the diagnosis, which included a combination of anti-EgP, anti-EgHF, anti-Em2+, and anti-EmG11 ELISA, as well as anti-AgB Western blot or EITB [30]. The anti-EmII/3-10 or anti-Em18 antibody ELISA results were used in this study as activity parameters [31,32,33]. The usefulness of anti-EmII/3-10 as a marker of parasite viability was first described in 2004 [31] and since then routinely used in our department. This test had not been performed in some patients of this cohort who were diagnosed with AE before 2004.

### 2.3. Analysis of Cross-Sectional Imaging

In order to determine differences in the number and size of AE lesions in the liver, contrast-enhanced cross-sectional imaging (CT, MRI, PET-CT) at or up to six months after diagnosis was analyzed using the software IMPAX EE R20 XVIII SU1 (AGFA Healthcare, Mortsel, Belgium) Version 20190821_0813. Extrahepatic lesions were not included in this analysis. As a surrogate marker for lesion size, the maximal diameter in the axial plane during the portal-venous phase was used. Individual lesions were defined as lesions completely surrounded by liver tissue or capsule. Conglomerates of lesions were, therefore, measured as one lesion if the individual lesions were not separated by liver tissue. In the case of multiple lesions, the sum of all maximal diameters was calculated, and the mean lesion size was calculated by dividing the sum of maximal diameters by the number of lesions.

### 2.4. Delay of AE Diagnosis

The time between first imaging (US, CT, MRI, PET-CT) describing a suspicious lesion and diagnosis of AE either through additional positive serology, histopathology, or PCR from biopsy, was calculated in days to determine the delay of diagnosis. 

### 2.5. Statistical Analysis

Statistical analysis was performed using GraphPad Prism 8 (GraphPad Software, Inc., San Diego, CA, USA) Version 8.0.0 (224) and IBM SPSS Statistics (IBM, Armonk, NY, USA) Version 26.0.0.0. To analyze the number of new AE cases per year and compare slopes between groups linear regression was used. To compare differences in nonparametric, unpaired, numerical data between groups the Mann–Whitney U test was used. For unpaired, categorical data the chi-square test was applied. A two-tailed *p*-value < 0.05 was regarded as statistically significant. 

### 2.6. Ethical Approval

In November 2020, ethical approval was obtained for the Zurich Echinococcosis Cohort Study (ZECS) from the local ethics committee (EC) in Zurich (Kantonale Ethikkommission Zürich, EC ZH, BASEC: 2020-00495). Patients who were still followed up at our clinic provided written informed consent. For those that had died, written informed consent was waived. Ethical approval for this study was additionally obtained from the local ethics committee (EC) in Zurich (Kantonale Ethikkomission Zürich, EC ZH, BASEC ID:2022-00085).

## 3. Results

### 3.1. Total AE Cases and Immunosuppression-Associated Conditions

Of 227 patients who were treated for alveolar echinococcosis at our center between 2000 and 2021, 189 patients consented to participation in the Zurich Echinococcosis Cohort Study (Figure 1). Of these 189 study participants, 38 also had one or more immunosuppression-associated condition at or up to 1 year after diagnosis (Figure 1). The reported IACs comprised malignancy (*n* = 15), chronic inflammatory disease (*n* = 11), diabetes mellitus (*n* = 8), cirrhosis (*n* = 4), transplantation (*n* = 2), HIV/AIDS (*n* = 1), and medication (*n* = 20) (Table 1). Reported malignancies were breast cancer (*n* = 4), prostate cancer (*n* = 2), colorectal cancer (*n* = 2), ovarian cancer (*n* = 1), nonsmall cell lung cancer (*n* = 1), parotid gland cancer (*n* = 1), carcinoid (*n* = 1), medullary thyroid cancer (*n* = 1), myelofibrosis (*n* = 1) and Morbus Waldenström (*n* = 1) (Table 1). The reported chronic inflammatory diseases consisted of rheumatoid arthritis (*n* = 3), chronic polyarthritis (*n* = 3), polymyalgia rheumatic (*n* = 2), psoriasis (*n* = 1), vasculitis (*n* = 1), and collagenosis (*n* = 1) (Table 1). In four patients, cirrhosis Child–Pugh A (*n* = 1), B (*n* = 2), and C (*n* = 1) were reported (Table 1). The immunosuppressive medication comprised conventional chemotherapy, including 5-FU, capecitabine, oxaliplatin, carboplatin paclitaxel, pemetrexed, raltitrexed, rituximab and bendamustine, as well as tacrolimus, mycophenolate, leflunomide, methotrexate, anti-TNFa inhibitors, tofacitinib and prednisone (Table 1). Chronic kidney disease and hereditary immunodeficiencies were not reported in any cohort patient.

### 3.2. New AE Cases per Year

**Figure 2:**
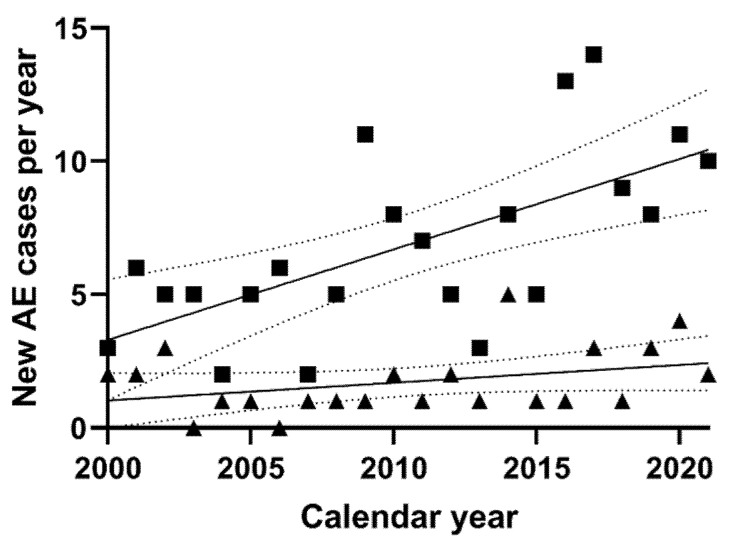
New AE diagnosis by calendar year. Shown are the number of new AE diagnoses by calendar year. Patients without an IAC are represented by squares, and those with an immunosuppression-associated condition, by triangles. The fitted linear regression curves are displayed as solid lines and their 95% confidence bands by dotted lines. Linear regression shows a significant increase in patients without an IAC over the years (y = 0.3394x − 675.4, *p* = 0.001). On the contrary, while there is a trend of an increasing number of newly diagnosed AE patients with an IAC per year (y = 0.06663x − 132.2), the slope is not statistically nonzero (*p* = 0.112).

From 2000 to 2021, a significant increase in all new AE cases per year could be observed by linear regression analysis (y = 0.4060x − 807.6, *p* = 0.001, data not shown). However, while linear regression of new AE cases with an IAC demonstrated a slightly positive slope (y = 0.06663x − 132.2), this was not significantly nonzero (*p* = 0.112, Figure 2). On the contrary, linear regression of new AE cases without an IAC demonstrated a more prominent positive slope (y = 0.3394x − 675.4), which was significantly nonzero (*p* = 0.001) and significantly different from the slope of patients with an IAC (*p* = 0.008, Figure 2).

### 3.3. Clinical Characteristics at AE Diagnosis

Serologic testing for echinococcosis was positive in all patients but four patients, two each in the group with an IAC (5.3%) and the group without an IAC (1.3%; data not shown). These cases were, however, confirmed by biopsy in the two IAC patients or after surgical resection in the non-IAC patients. In six cases, two with an IAC (5.3%) and four without (2.6%), only enzyme-linked immunoelectrotransfer blot (EITB) or Western blot were diagnostic (data not shown). Thirteen patients, four with an IAC and nine without, had liver biopsies performed prior to or despite positive serology for echinococcosis (data not shown). In 70 patients, the diagnosis was confirmed on histopathology after surgical resection.

Between the group of patients with and without IACs, there was a significant difference regarding age at diagnosis, with the patients with IACs being significantly older (Table 2). While there was no significant difference between the individual P, N, and M classifications, patients with an IAC were diagnosed significantly more often in an earlier stage (Table 2). The number of lesions did not differ significantly (Table 2): 54% without and 47% with an IAC had a single lesion (data not shown). Patients with an IAC tended to have smaller cumulative lesion sizes, but this difference was not significant (Table 2). However, patients with an IAC demonstrated significantly smaller mean lesion size and significantly less frequently a positive anti-Em18 ELISA result at diagnosis (Table 2). In these patients, AE was significantly more often an incidental finding, although the diagnosis was slightly but significantly delayed, compared with patients without an IAC (Table 2). There were no differences in the number of patients that underwent radical surgery (Table 2). 

### 3.4. Clinical Course of AE Patients with an IAC

The median follow-up time of patients with an IAC was 54 months, ranging from 1 to 234 months (Table 3). Of the 38 patients with an IAC, 14 had surgical resection of the AE lesion, and one received liver transplantation for decompensated liver cirrhosis. In eight of these patients, the benzimidazole therapy could be stopped after two years (Table 3). None showed recurrent disease after a median follow-up of 71.5 months, with the shortest follow-up of a patient off therapy of 32 months (Table 3). In 16 patients, surgical resection was not pursued due to patient and physician choice. Of the 24 that did not receive surgical resection of the AE lesion, two showed slightly progressive lesions on the last cross-sectional imaging during a median follow-up of 47.5 months (Table 3). The reported increase in maximal diameter was 9 mm and 3 mm, respectively (data not shown). However, the first of these two patients had not been on benzimidazole therapy due to intolerance for 6.5 years (inoperable), and the second had only been on benzimidazole therapy for 9 months at the time of follow-up imaging.

## 4. Discussion

In the current study, we confirmed earlier observations of increasing numbers of new AE cases in different European centers [7,8,9,10]. Over the entire period, in a fifth of all patients, an IAC was reported at or shortly after the AE diagnosis. However, we showed that the increase in new AE cases per year was mostly among patients without an IAC. We also showed that AE patients with an IAC were older at diagnosis and more frequently diagnosed incidentally with AE at an earlier stage with smaller mean lesion size and less frequently anti-Em18 positive ELISA results. The latter could be an effect of the earlier stage and smaller lesion size, as anti-Em18 serology has been shown to correlate with both [34,35]. Since PET imaging was not routinely performed at diagnosis, it remains unknown whether these patients had inactive diseases that might otherwise have never been diagnosed. These results implicate that increased use of imaging in this population may lead to an earlier diagnosis of AE, but this remains speculative.

Furthermore, patients with IACs showed a favorable clinical course, with no recurrences after surgery, and a stable disease course was established in all but two inoperable cases, where other reasons for disease progression have to be considered, such as benzimidazole intolerance and short therapy duration. Altogether, the results of this study lead us to the conclusion that IACs have little impact on the rising number of AE cases, as well as the characteristics of AE at diagnosis and its clinical course. Conversely, we hypothesize that it is most likely the underlying immunosuppressive conditions that lead to more frequent cross-sectional imaging and, therefore, earlier detection of AE. While IACs could possibly contribute to an accelerated disease course, if AE was left untreated, the availability of potent medical therapy with benzimidazoles reduces their significance in regard to the AE’s clinical course.

When comparing these results to previous findings, including those by Chauchet et al. [8] and Lachenmayer et al. [10], there are differences and similarities between our study and those of others that have to be addressed. First, Chauchet et al. did not include patients with chronic kidney or liver disease, or diabetes, due to the reasoning that this could lead to an overestimation of the effect of immunosuppression in AE [8]. Conversely, Lachenmayer et al. also included patients with asthma and other unspecified immunosuppressive conditions [10]. We argue that differences in the degree of immunosuppression cannot be classified by underlying disease entity, since even within a single entity the degree of immunosuppression can vary depending on disease activity and or stage, as well as the medical treatment applied. Furthermore, it is unknown whether there are differences in these conditions and the extent of immunosuppression regarding the risk of attracting AE. Therefore, we chose an inclusive definition of immunosuppression-associated conditions and, by doing so, found little impact of IACs on the increase in new AE diagnoses. Other relevant differences are the time frame and type of analysis that we chose to investigate an increase in AE patients with an IAC. Both mentioned studies evaluated AE patients whose diagnosis dates back to the 1980s and 1970s and found most cases with an IAC since the new millennium [8,10]. In contrast, we evaluated patients from 2000 onward and chose this timeframe because we expected optimal data quality from these patients, as they were all included in our center’s electronic hospital information system KISIM, which stores reports of all clinics at the center and correspondences with other hospitals, as well as imaging reports and the individual image data. While the previous studies showed significant increases in pooled cases during specified time periods of 10 years, our analysis shows no significant increase in linear regression of annual cases over 21 years [8,10]. Finally, the similarities of our study findings to those of Chauchet et al. are that in patients with an IAC, the diagnosis was more frequently incidental and in an earlier stage with negative specific serology [8]. 

Naturally, there are limitations to this study. These include the patients who had to be excluded due to missing consent, the retrospective nature of data analysis, and missing data in both groups. Furthermore, the absolute number of patients with an IAC was small, even though a broad definition of IACs was applied. This could be the reason why certain analyses did not achieve statistical significance, for instance, in cumulative lesion size and PNM classification. Altogether, however, for a rare disease such as AE, the total number of study participants is significant, and the findings between different analyses are conclusive. Finally, since the population at risk over the studied time period is impossible to determine, it remains unclear whether patients with an IAC are at increased risk for developing AE after exposure to *E. multilocularis* eggs, compared with the healthy population.

## 5. Conclusions

Since 2000, IACs have had little impact on the increase in new AE cases, as well as on the extent of the disease at diagnosis and clinical course of AE.

## Figures and Tables

**Table 1:** Composition of immunosuppression-associated conditions.

Malignancy (15)	Breast cancer (4)
	Prostate cancer (2)
	Colorectal cancer (2)
	Ovarian cancer (1)
	Non-small cell lung cancer (1)
	Parotid gland cancer (1)
	Carcinoid (1)
	Medullary thyroid cancer (1)
	Myelofibrosis (1)
	Morbus Waldenström (1)
Chronic inflammatory disease (11)	Rheumatoid arthritis (3)
	Chronic polyarthritis (3)
	Polymyalgia rheumatica (2)
	Psoriasis (1)
	Vasculitis (1)
	Collagenosis (1)
Diabetes mellitus (8)	Type I (1)
	Type II (7)
Cirrhosis (4)	Child–Pugh A (1)
	Child–Pugh B (2)
	Child–Pugh C (1)
Organ transplantation (2)	Heart (1)
	Liver (1)
HIV/AIDS (1)	AIDS (1)
Medication (20)	Chemotherapy (8)
	Immunosuppressive/anti-inflammatory therapy (12)

Shown are the different immunosuppression-associated conditions that were reported in patients at or up to a year after diagnosis of AE. The pie chart reflects the composition of disease entities in the study and not of individual patients. Patients could have one or more IACs.

**Table 2:** Characteristics of AE at diagnosis.

	Immunocompetent (*n* = 151)	Immunosuppression-Associated Conditions (*n* = 38)	*p*-Value (Univariate)
Age at diagnosis	55 y; 12–80 y	60.5 y; 39–77 y	0.002 *
Sex	82 f (54.3%)	21 f (55.3%)	0.916
	69 m (45.7%)	17 m (44.7%)	
P	I: 61 (40.4%)	I: 22 (57.9%)	0.126
	II: 28 (18.5%)	II: 7 (18.4%)	
	III: 36 (23.8%)	III: 3 (7.9%)	
	IV: 25 (16.6%)	IV: 5 (13.2%)	
	0: 1 (0.7%)	0: 1 (2.6%)	
N	0: 113 (74.8%)	0: 34 (89.5%)	0.052
	I: 37 (24.5%)	I: 3 (7.9%)	
	X: 1 (0.7%)	X: 1 (2.6%)	
M	0: 134 (88.7%)	0: 35 (92.1%)	0.547
	I: 17 ^#^ (11.3%)	I: 3 ^##^ (7.9%)	
Stage	I: 42 (27.8%)	I: 21 (55.3%)	0.015 *
	II: 20 (13.2%)	II: 6 (15.8%)	
	IIIa: 25 (16.6%)	IIIa: 3 (7.9%)	
	IIIb: 39 (25.8%)	IIIb: 4 (10.5%)	
	IV: 25 (16.6%)	IV: 4 (10.5%)	
Pos. anti-Em18 ELISA	Yes: 70 (46.4%)	Yes: 10 (26.3%)	0.034 *
	No: 61 (40.4%)	No: 21 (55.3%)	
	Missing: 20 (13.2%)	Missing: 6 (15.8%)	
Incidental finding	Yes: 60 (39.7%)	Yes: 30 (78.9%) No: 8 (21.1%)	0.000 *
	No: 84 (55.6%)		
	Missing: 7 (4.6%)		
Delay of diagnosis	16 d, 2–3954 d	26.5 d, 2–650 d	0.030 *
	Missing: 10 (6.6%)		
Lesion count	1; 1–38	1; 1–17	0.555
	Missing: 7 (4.6%)	Missing: 4 (10.5%)	
Lesion size, cum.	103 mm; 15–792 mm	86 mm; 13–390 mm	0.052
	Missing: 7 (4.6%)	Missing: 4 (10.5%)	
Lesion size, avg.	64 mm; 9–213 mm	44 mm; 8–151 mm	0.036 *
	Missing: 7 (4.6%)	Missing: 4 (10.5%)	
Surgical resection	56 (37.1%)	14 (34.2%)	0.742

Shown are the characteristics of all 189 patients at diagnosis, divided into two groups, depending on whether they did or did not have an immunosuppression-associated condition. Numerical data are shown as median and range. * *p*-values < 0.05 were regarded as statistically significant. y = years, d = days, m = male, f = female, mm = millimeters. ^#^ lung (*n* = 5), peritoneum (*n* = 3), lung and brain (*n* = 2), brain (*n* = 2), spleen and peritoneum (*n* = 1), retroperitoneum (*n* = 1), adrenal gland (*n* = 1) ^##^ lung (*n* = 2), spleen and peritoneum (*n* = 1).

**Table 3:** Follow-up of patients with an immunosuppression-associated condition.

ID	Age	Sex	PNM	Stage	Em18	IF	L.c.	L.s.c.	L.s.a.	Res.	Tfu	Prog.	IAC (Disease)	IAC (Medication)
1	58	f	P1N0M0	I	n.a.	yes	n.a.	n.a.	n.a.	yes ^#^	169	no	CRC	5FU, raltitrexed
2	69	m	P1N0M0	I	-	yes	1	28	28	no	42	no	CPA	MTX, prednisone
3	71	f	P1N0M0	I	n.a.	yes	n.a.	n.a.	n.a.	yes	80	no	RA	MTX
4	59	m	P3N0M0	IIIa	+	yes	1	91	91	yes	62	no	DM II	---
5	61	m	P4N1M0	IV	n.a.	no	1	141	141	no	230	no	Psoriasis	---
6	39	m	P3N0M0	IIIa	n.a.	yes	1	91	91	no	234	no	AIDS	---
7	75	f	P1N0M0	I	n.a.	yes	n.a.	n.a.	n.a.	yes ^#^	54	no	PMR	prednisone
8	46	m	P1N0M0	I	n.a.	yes	1	37	37	yes ^#^	171	no	Cirrhosis Child C	---
9	68	f	P2N0M0	II	+	yes	1	85	85	no	43	no	Vasculitis	prednisone
10	51	m	P2N0M0	II	+	n.a.	1	73	73	no	40	no	BrCa	---
11	44	f	P4N0M1	IV	-	no	3	196	65	no	161	no	CPA	prednisone
12	67	f	P1N0M0	I	-	yes	1	13	13	yes	133	no	BrCa	---
13	48	m	P1N0M0	I	+	yes	3	58	19	no	128	no	Cirrhosis Child B	---
14	71	m	P2N1M1	IV	-	no	4	149	37	no	137	no	RA, DM II	MTX, prednisone
15	67	f	P2N0M0	II	-	yes	1	84	84	yes ^#^	113	no	DM I	---
16	60	m	P4N0M0	IIIb	+	no	1	125	125	no	107	no	PGCa	---
17	54	f	P3N0M0	IIIa	+	yes	1	151	151	yes	73	no	Myelofibrosis	---
18	65	f	P1N0M0	I	-	yes	2	74	37	yes ^#^	92	no	Cirrhosis Child A	---
19	63	f	P1N0M0	I	-	yes	1	46	46	no	89	yes *	PMR	prednisone
20	66	f	P1N0M0	I	-	yes	11	93	8	no	86	no	DM II	---
21	55	f	P2N0M0	II	+	yes	1	57	57	no	35	no	mThyCa	---
22	76	m	P1N1M0	IIIb	-	no	1	126	126	no	21	no	CPA	leflunomid, MTX
23	65	m	P1N0M0	I	-	yes	4	125	31	no	74	no	heart TPL	tacrolimus, MMF, prednisone
24	61	f	P2N0M0	II	-	no	1	55	55	yes ^#^	70	no	BrCa	unknown CTx-agent
25	54	f	P1N0M0	I	-	yes	7	91	13	no	54	no	BrCa	unknown CTx-agent
26	60	f	P1N0M0	I	+	yes	5	117	23	no	54	no	CRC	oxaliplatin, capecitabine
27	55	m	P1N1M0	IIIb	+	no	2	87	44	no	52	no	Carcinoid	oxaliplatin
28	55	f	P1N0M0	I	-	yes	2	87	44	yes	31	no	RA	anti-TNFa, tofacitinib
29	58	f	P1N0M0	I	-	yes	1	45	45	no	41	no	DM II	---
30	67	f	P1N0M0	I	-	no	2	115	58	yes ^#^	32	no	liver TPL ^##^	tacrolimus, MMF
31	69	m	P4N0M0	IIIb	+	no	17	390	23	no	28	no	PrCa	---
32	77	f	P4N0M0	IIIb	-	yes	2	114	57	no	1	n.a.	Collagenosis	prednisone
33	61	f	P1N0M0	I	-	yes	2	84	42	yes	16	no	OvCa	carboplatin, paclitaxel
34	60	m	P1N0M0	I	-	yes	6	84	14	no	20	no	DM II	---
35	64	m	P1N0M0	I	-	yes	3	40	13	no	9	yes **	DM II	---
36	60	f	P1N0M0	I	-	yes	1	29	29	no	8	no	Waldenström	rituximab, bendamustin
37	67	m	PxNxM1	IV	-	yes	n.a.	n.a.	n.a.	yes ^#^	8	no	NSCLC, Cirrhosis Child B, DM II	carboplatin, permetrexed
38	58	m	P2N0M0	II	-	yes	1	64	64	no	6	no	PrCa	---

Depicted are all patients with reported immunosuppression-associated condition (IAC) with age at diagnosis, gender, PNM classification and stage, anti-Em18 serology, AE lesion count (L.c.), cumulative AE lesion size (L.s.c), average AE lesion size (L.s.a.), reported AE-specific symptoms (Sym.), surgical resection (Res.), follow-up time (Tfu) in months, reported disease progression (Prog.). AIDS = acquired immune deficiency syndrome, BrCa = breast cancer, CPA = chronic polyarthritis, CRC = colorectal cancer, DM = diabetes mellitus, NSCLC = nonsmall cell lung cancer, OvCa = ovarian cancer, PGCa = parotid gland cancer, PMR = polymyalgia rheumatica, PrCa = prostate cancer, RA = rheumatoid arthritis, mThyCa = medullary thyroid cancer, TPL = transplantation. * intolerance to BMZ therapy, ** only 9 months of BMZ therapy. ^#^ benzimidazole therapy was stopped two years after surgery. ^##^ de novo infection, a CT 2 months post-liver transplant showed no AE lesion.

## Data Availability

Data cannot be shared publicly because of confidentiality reasons. Data are available through the Ethics Committee Zurich (contact via info.kek@kek.zh.ch) for researchers who meet the criteria for access to confidential data.

## Abbreviations

AE	Alveolar echinococcosis
CLL	Chronic lymphocytic leukemia
CT	Computed tomography
CTx	Chemotherapy
DNA	Deoxyribonucleic acid
EITB	Enzyme-linked immunoelectrotransfer blot
ELISA	Enzyme-linked immunosorbent assay
FDG	Fluorodeoxyglucose
IAC	Immunosuppression-associated condition
MRI	Magnetic resonance imaging
NLR	Neutrophil-to-lymphocyte ratio
PET	Positron emission tomography
STROBE	Strengthening the Reporting of Observational Studies in Epidemiology
US	Ultrasound
ZECS	Zurich Echinococcosis Cohort Study
